# Signaling Network Map of Endothelial TEK Tyrosine Kinase

**DOI:** 10.1155/2014/173026

**Published:** 2014-10-13

**Authors:** Aafaque Ahmad Khan, Varot K. Sandhya, Priyata Singh, Deepak Parthasarathy, Awinav Kumar, Jayshree Advani, Rudrappa Gattu, Dhanya V. Ranjit, Rama Vaidyanathan, Premendu Prakash Mathur, T. S. Keshava Prasad, F. Mac Gabhann, Akhilesh Pandey, Rajesh Raju, Harsha Gowda

**Affiliations:** ^1^Institute of Bioinformatics, International Tech Park, Whitefield, Bangalore 560066, India; ^2^School of Biotechnology, KIIT University, Bhubaneswar 751024, India; ^3^Dr. M.G.R. Educational and Research Institute, Maduravoyal, Chennai 600095, India; ^4^Manipal University, Madhav Nagar, Manipal 576104, India; ^5^Department of Biomedical Engineering and Institute for Computational Medicine, Johns Hopkins University, Baltimore, MD 21218, USA; ^6^McKusick-Nathans Institute of Genetic Medicine, Johns Hopkins University School of Medicine, Baltimore, MD 21205, USA; ^7^Department of Biological Chemistry, Johns Hopkins University School of Medicine, Baltimore, MD 21205, USA; ^8^Department of Pathology, Johns Hopkins University School of Medicine, Baltimore, MD 21205, USA; ^9^Department of Oncology, Johns Hopkins University School of Medicine, Baltimore, MD 21205, USA

## Abstract

TEK tyrosine kinase is primarily expressed on endothelial cells and is most commonly referred to as TIE2. TIE2 is a receptor tyrosine kinase modulated by its ligands, angiopoietins, to regulate the development and remodeling of vascular system. It is also one of the critical pathways associated with tumor angiogenesis and familial venous malformations. Apart from the vascular system, TIE2 signaling is also associated with postnatal hematopoiesis. Despite the involvement of TIE2-angiopoietin system in several diseases, the downstream molecular events of TIE2-angiopoietin signaling are not reported in any pathway repository. Therefore, carrying out a detailed review of published literature, we have documented molecular signaling events mediated by TIE2 in response to angiopoietins and developed a network map of TIE2 signaling. The pathway information is freely available to the scientific community through NetPath, a manually curated resource of signaling pathways. We hope that this pathway resource will provide an in-depth view of TIE2-angiopoietin signaling and will lead to identification of potential therapeutic targets for TIE2-angiopoietin associated disorders.

## 1. Introduction

Angiopoietin-TIE2 is one of the major signaling systems that regulates development and remodeling of vascular system [1, 2]. TIE2 is a member of the TIE receptor tyrosine kinase family that is preferentially expressed in endothelial cells [3]. Among the angiopoietins (angiopoietin-1, angiopoietin-2, and angiopoietin-4 in humans), angiopoietin-1 (ANGPT1) is known as a constitutive agonist of TIE2. ANGPT1/TIE2 signaling promotes endothelial cell survival, endothelium integrity, and anti-inflammatory/antiapoptotic responses supporting reduced vascular permeability [4, 5]. ANGPT2 is generally considered as antagonist as it competes with ANGPT1 for binding to TIE2, reduces vessel stability, and enhances vascular remodeling [6]. However, under specific experimental conditions, ANGPT2 has been shown to promote endothelial-cell survival, sprouting, and migration in a temporal and concentration-dependent manner [7–9]. Therefore, angiopoietin-2 (ANGPT2) is currently considered as a context dependant agonist or antagonist of TIE2 [6, 10]. Angiopoietin-4 (ANGPT4) is also known to be an agonist of TIE2 while angiopoietin-3 (ANGPT3), the mouse ortholog of angiopoietin-4, is reported to be antagonistic to TIE2 [11]. The other member of the TIE family is the orphan receptor TIE1. It heterodimerizes with TIE2 and modulates TIE2 signaling induced by ANGPT1 and ANGPT2 [12]. ANGPT1 binding to TIE2 induces dissociation of the TIE1-TIE2 complex [12]. This suggests that TIE2 signaling is regulated by the molecular balance between ANGPT1 and ANGPT2 [6, 13] and TIE1 and TIE2, with another one being the ectodomain cleavage of TIE receptors [14]. The activation of TIE2 is achieved by the assembly with tetrameric or higher order multimeric angiopoietins, clearly differentiating TIE2 from other tyrosine kinase receptors [15]. ANGPT1 induces the translocation of TIE2 to cell-cell junctions and transassociation in the form of homomeric complexes to activate the downstream signaling of TIE2 [16].

Binding of ANGPT1 to TIE2 leads to receptor dimerization and subsequent activation followed by autophosphorylation at specific tyrosine residues [15, 17]. These phosphorylated sites provide binding platform to a number of effector molecules to initiate downstream signaling cascade which ultimately controls various cellular responses including morphogenesis, proliferation, extracellular matrix interaction, permeability, survival, and differentiation [18–23]. TIE2 interacts with p85 subunit of phosphatidylinositol-3-kinase (PI3K)* via* Tyr-1101 and activates PI3K-AKT pathway which inhibits Smac release from mitochondria and increases the expression of survivin leading to survival and chemotaxis of endothelial cells [18, 24, 25]. AKT activation also inhibits forkhead transcription factor FKHR (FOXO1) which protects endothelial cells from apoptosis [26]. ANGPT1 also induces the PI3K/AKT mediated activation of eNOS and NO release in endothelial cells [27, 28]. In endothelial cells, both ANGPT1 and ANGPT2 also induce TIE2-dependent translocation of P-selectin through a PLCG1/Ca2+ signaling pathway [29].

SH2 domain containing proteins such as growth factor receptor-bound protein 2 (GRB2), growth factor receptor-bound protein 7 (GRB7), growth factor receptor-bound protein 14 (GRB14), protein tyrosine phosphatase nonreceptor type 11 (SHP-2), and phosphoinositide-3-kinase (PI3K) is recruited and transphosphorylated by TIE2 [30]. GRB2 and SHC1 recruit SOS1 and lead to the activation of Ras-Raf-mitogen activated protein kinase (MAPK) pathway that regulates platelet activating factor synthesis, anti-inflammatory responses, and endothelial cell migration, proliferation, permeability, and morphogenesis [5, 20–22, 31, 32]. Through SOS1 or PI3Ks, angiopoietin/TIE2 system also regulates the activation of RAC1, RHOA, CDC42, and focal adhesion kinase 1 to mediate cytoskeleton reorganization and migration of endothelial and synovial cells [33]. Angiopoietin-1 induced activation of RHOA results in sequestration of SRC by DIAPH1 thereby preventing SRC association with VEGFR2 [34]. Recruitment of dynamic complexes comprising NCK adaptor protein 1 (NCK1), RAS p21 protein activator 1 (p120GAP), and P21 protein-activated kinase 1 (PAK1), to TIE2 by the DOKs, especially DOK2, has been attributed to increased cell motility [35]. TIE2 also interacts with the inhibitor of nuclear factor kappa B (NF-kB) activity TNFAIP3 interacting protein 2 (ABIN-2) that inhibits NF-kB transcriptional activity and mediates anti-inflammatory and antiapoptotic action [36, 37]. TIE2 activation induces the phosphorylation of STAT1, STAT3, and STAT5A/5B and their subsequent translocation into nucleus to induce the expression of the cell cycle inhibitor cyclin-dependent kinase inhibitor 1A (p21) [38]. ANGPT2 also interacts with integrins like integrin αV*β*5, αV*β*3, and α5*β*1 in endothelial cells with less affinity than TIE2 and can induce TIE2-independent signaling [39]. TIE2 also forms a complex with α5*β*3 and FAK. ANGPT2 induces phosphorylation of FAK at Serine910, α5*β*3 internalization, and dissociation of p130CAS and talin from α5*β*3 [40]. Recently, ANGPT2 has also been shown to induce the activation of ERK/MSK1/CREB pathway to impart cell survival and resistance to doxorubicin in HepG2 cells [41].

Besides the defects in vascular system and angiogenesis [42–44], TIE2 signaling has also been associated with rheumatoid arthritis [45] and asthma [46]. Considering the importance of TIE2 signaling, here we provide a manually curated enhanced network map of angiopoietin(s)-induced TIE2-mediated signaling events as a reference platform for further biomedical investigations.

## 2. Methods

We screened published research articles related to TIE2 signaling. NetPath criteria described earlier [47, 48] were followed for the annotation of protein-protein interactions (PPIs), enzyme-substrate relationships, and posttranslational modifications (PTMs) (catalytic events). Activation/inhibition status of proteins, alterations in protein localization, and also genes regulated at mRNA level by TIE2 signaling were also documented. PathBuilder, an in-house pathway annotation tool, was used for the curation of these reactions [49]. Each curated reaction was internally reviewed by trained biocurators followed by an external review by a Pathway Authority, an expert in the field (FMG, coauthor of this paper).

## 3. Results and Discussion

Our analysis resulted in the cataloging of 140 unique molecules that are reported in TIE2 signaling. These molecules were part of 43 PPIs and 102 catalytic events, 23 activation/inhibition events, and 11 protein translocation events. We have also documented 124 and 65 genes that were reported to be upregulated and downregulated, respectively, by TIE2 signaling in response to angiopoietin(s) in human cells. The curated data for TIE2 signaling pathway is freely available to the scientific community for visualization and download in different community standard data exchange formats through NetPath [http://www.netpath.org/], a resource of signaling pathways. These formats include Proteomics Standards Initiative for Molecular Interaction (PSI-MI version 2.1), Biological Pathway Exchange (BioPAX level 3), and Systems Biology Markup Language (SBML level 2.1).

**Figure 1:**
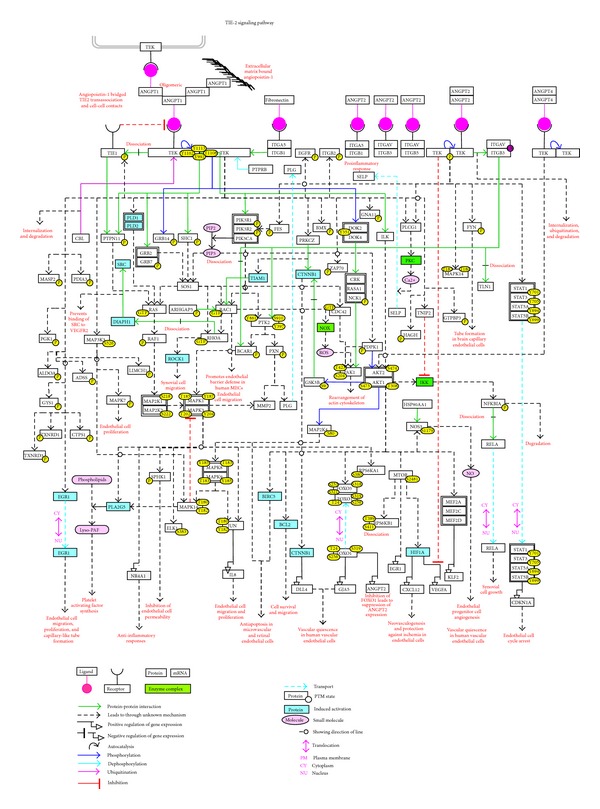
A detailed map of TIE2 signaling. This is a manually drawn (using PathVisio) pictorial representation of the network of reactions annotated in NetPath. The topology of the molecules and their reactions from TIE2 to the transcription factors are derived from the experimental information obtained by the use of inhibitors, activators, mutants, and silencing approaches. Each node represents the molecules and the edges represent the relationships between them as provided in the figure legend.

For effective visualization, we have graphically represented the reactions and various cellular processes that those reactions mediate in the context of specific studies on TIE2 signaling (Figure 1). PathVisio, an open visualization tool was used to manually depict this information [50]. The pathway map can also be accessed through NetSlim (http://www.netpath.org/netslim/TIE2_pathway.html), a resource that provides a smaller version of the pathway by filtering data based on predefined confidence threshold criteria [51]. At NetSlim, a “map with citation” is also provided in which each reaction is linked to the corresponding literature through PubMed. Users can download these maps in customizable formats such as GenMAPP and gpml.

## 4. Conclusions

This open-access pathway data enables better analysis of high-throughput experimental data and hypothesis-driven approaches to study the dynamics of TIE2 signaling for therapeutic interventions. Information on TIE2 pathway in NetPath will be periodically updated to reflect novel findings relevant to TIE2 signaling. We intend to provide information pertaining to cross-talks of other ligand/receptor systems such as VEGF, TNF-alpha, and integrins with TIE2 and* vice versa*, in the subsequent versions in NetPath. We encourage scientific community to help us maintain this resource up-to-date and error-free through http://www.netpath.org/comments.
